# A Case Study on Polypharmacy and Depression in a 75-Year-Old Woman with Visual Deficits and Charles Bonnet Syndrome

**DOI:** 10.3390/geriatrics7010005

**Published:** 2021-12-28

**Authors:** José Caamaño-Ponte, Martina Gómez Digón, Mercedes Pereira Pía, Antonio de la Iglesia Cabezudo, Margarita Echevarría Canoura, David Facal

**Affiliations:** 1 Grupo de Investigación Dependencia, Gerontología, y Geriatría, Universidade Santiago de Compostela, 15782 Santiago de Compostela, Spain; caamajos@hotmail.com; 2 Servicio Enfermería EOXI Lugo, 27003 Lugo, Spain; marttina24@gmail.com; 3 Servicio Farmacia EOXI Lugo, 27003 Lugo, Spain; Maria.Mercedes.Pereira.Pia@sergas.es (M.P.P.); antonioluis.de.la.iglesia.cabezudo@sergas.es (A.d.l.I.C.); 4 Sanitas Hospitales A Coruña, 15005 A Coruna, Spain; mechevarriac@sanitas.es; 5 Departamento de Psicología Evolutiva y de la Educación, Universidade de Santiago de Compostela, 15782 Santiago de Compostela, Spain

**Keywords:** depression, adjustment disorder, glaucoma, hallucinations, polypharmacy

## Abstract

Depression is one of the most prevalent pathologies in older adults. Its diagnosis and treatment are complex due to different factors that intervene in its development and progression, including intercurrent organic diseases, perceptual deficits, use of drugs, and psycho-social conditions associated with the aging process. We present the case of a 75-year-old woman (who lives in the community) with a diagnosis of major depression with more than 10 years of history, analyzing her evolution and therapeutic approach.

## 1. Introduction

Depressive disorders are the most common psychiatric pathology in old adulthood. It is associated with various mental and biological stressors that affect the functional capacity and independence of old adults, reducing their quality of life. International studies show variable prevalences that range between 8.8% and 23.6% in Europe [1,2], could reach 60% in Latin America, and would exceed 38% in rural Asian populations. This geographical variability is due to methodological, clinical, and sociocultural differences. Recent studies in Spain inform that up to 36% of older people living in urban areas in the community suffer from depression [3,4,5]. Depression seems to be more frequent in the female sex. However, this observation could be biased because women present a greater longevity and/or a greater tendency to go to medical services than men, whereas men present a more severe somatic expression of psychiatric symptoms and/or a higher reluctance to express psychiatric symptoms than women [6].

Depressive symptoms include affective disorders such as sadness, apathy, emotional lability and crying, anhedonia, and nihilism; behavior modifications such as anxiety, irritability, insomnia, and hyporexia; and alterations in the course and content of thought, as well as cognitive and physical frailty. Among all the symptoms, autolytic ideation requires a specific comment, since depression is the main suicide risk factor in old age. Suicide constitutes one of the 10 main causes of death in the old adults, mainly in men aged 65 and over who use more lethal methods conditioned by loneliness and isolation [7,8]. In old populations, the etiology of depression is multifactorial: there are psychosocial causes derived from the aging process (family losses, work life, loneliness, environmental barriers, lack of resources, lack of social support) in addition to genetic and biological factors that contribute to the increase in frailty, geriatric syndromes, and dependency [9,10].

Its diagnosis is clinical, following the criteria included in the International Classification of Diseases (ICD-10) or the Diagnostic and Statistical Manual of Mental Disorders (DSM-5, APA 2014). In old adults, the diagnosis can be complex due to comorbidity and drugs that potentially induce psychiatric symptoms and iatrogenic complications, to adaptive disorders following age-related changes and/or to incipient cognitive impaiments. In any case, it can be an underdiagnosed disease due to circumstances related to its own nature, personality factors, and, also, because of the peculiarities of the healthcare systems [11].

To optimize its treatment, a transdisciplinary approach is required based on antidepressant drugs, such as selective serotonin reuptake inhibitors (SSRIs) or serotonin and norepinephrine reuptake inhibitors (SSNRIs), which have shown different therapeutic efficacy. It may also require, in many cases, mood stabilizers, anxiolytics, antipsychotics, tricyclic antidepressants (TCAs), or monoamine oxidase inhibitors (MAOIs) [12,13,14], plus psychosocial approaches (cognitive-behavioral psychotherapy, supportive psychotherapy), occupational techniques (re-education in activities of daily living, training in the use of technical aids), and physical training, which help to improve the prognosis and prevent relapses [15].

Within the different age-related conditions that can interfire with the diagnosis of depression in old age, Charles Bonnet syndrome is characterized by the presence of complex visual hallucinations, triggered by vision deprivation in the absence of neurological, psychiatric, and/or systemic disorders. The patient usually perceives the hallucinations as not real, which reduces anxiety, although the content, duration, and frequency are variable. Charles Bonnet syndrome can be associated with age-related entities such as enucleation, optic neuritis, diabetic retinopathy, macular degeneration, cataracts, and glaucoma, among others. Accordingly, its prevalence is relatively high in geriatric patients. In patients with major depression, a differential diagnosis with psychotic disorders is required [16,17].

The main objective of the study has been to facilitate deliberation on the frequent interrelation between organic pathologies, depressive symptomatology, and their overlap in time in old patients, as well as to present the heterodox therapeutic approach in this case, taking into account the complexity of the health care model of the Autonomous Community of Galicia (northwest Spain) and the patient’s therapeutic choices.

## 2. Case

### 2.1. Personal History

A 75-year-old woman, who is right-handed, and a resident of the urban area of the province of A Coruña (Galicia, NW of Spain). She is married and has two children (one female and one male) and two male grandchildren. She lives with her husband (74 years old), who provides care support for the patient’s visual deficits. A medium education of schooling is possessed, along with an administrative profession and adequate social resources.

### 2.2. Ethical Standards

The study was conducted in accordance with the “Request for authorization for access and publication of health data as clinical case/case series” as provided in the General Data Protection Regulation (EU Regulation 2016/679 of the European Parliament and of the Council, 27 April 2016) and the Spanish regulations on personal data protection in force. Written informed consent was obtained from the participant. Due to visual deficits in the patient, the informed consent was read aloud and supervised by the caregiver.

### 2.3. Medical History

According to medical records, during this study the patient presented hypothyroidism, dyslipidemia, type II diabetes mellitus, macular degeneration, glaucoma, arterial hypertension, hypertensive heart disease, ChadsVasc4 persistent atrial fibrillation, extensive calcification of the mitral annulus, mild mitral regurgitation, moderate tricuspid regurgitation, lacunar stroke, vertigo, peripheral vascular disease, bronchial asthma, and acute bronchitis progressively diagnosed. The patient demonstrates no toxic habits.

The patient has been followed by the family medicine (FM) service of the center since the end of 2013, with the aim of carrying out a preventive approach, in coordination with doctors from other specialties such as cardiology, endocrinology, ophthalmology, neurology, and psychiatry.

In the initial clinical evaluation, previously diagnosed diseases were treated with levothyroxine sodium, Armolipid Plus, a nutraceutical based on berberine, red yeast, policosanols, coenzyme Q10, astaxanthin and folic acid, and Bimatoprost solution, to which clonazepam and duloxetine were added to treat anxiety-depression symptoms. The general physical examination showed no data of interest. A control analysis was requested, whose most significant results were glucose in serum/plasma 156 mg/dL, total cholesterol 300 mg/dL, HDL 48 mg/dL, LDL 232 mg/dL, TSH 0.93 mIU/L, and the need was emphasized for diet and physical exercise to adjust lipid levels, explaining that the patient ruled out lipid-lowering drug treatment due to fear of liver damage. The FM insisted on the convenience of carrying out a scheduled follow-up.

Between 2014 and 2018, the patient went to her FM and specialist doctors on different occasions to control her chronic diseases (mainly hypothyroidism, dyslipidaemia, Diabetes Mellitus, and Glaucoma). Acute diseases such as respiratory infection, viriasis, oral candidiasis, lump infectious breast, sciatica, or sacral-coccygeal trauma were successfully treated. She also received systematic immunization against the influenza virus. She underwent surgery for her visual pathology in 2016, with relative success and maintenance treatment consisting of Lutein, Bimatoprost, and Brinzolamide. Bronchial asthma with treated with Budesonide/Formoterol. Table 1 shows the main pharmacological treatment modifications made to date.

### 2.4. History of the Disease

The prevalent symptomatology referred to by the patient and her family throughout the depressive process consists of sadness, emotional lability and crying, low self-esteem, negativism, apathy, anxiety, insomnia, ruminant thinking, and occasional autolytic ideation. Regarding the loss of visual capacity and the secondary dependence to it, the diagnosis of glaucoma and macular degeneration has been subsequent to the onset of depressive symptoms.

Over the years, an evolution characterized by periods of emotional well-being with a significant reduction in symptoms and different relapses that required therapeutic adjustments has been observed. Monitoring of the depressive disorder is carried out by a psychiatrist outside the primary care center, who adjusts the psychotropic drugs periodically (Table 1).

From a non-pharmacological perspective, she was treated in the center’s psychology department. Psychologists detected family problems, poor socialization, and a lack of acceptance of the disease with reactivity to support proposals, such as technical aids for ambulation or functional independence. She also attended therapeutic programs of the Spanish National Organization for the Blind (ONCE), where she currently receives supportive psychotherapy and participates in activities such as gymnastics and choir. Regarding physical activity, ONCE provides cardiorespiratory and muscular maintenance as well as psychomotor coordination training.

### 2.5. Supplementary Tests

The patient’s multiple pathologies and her evolution have required the performance of different complementary tests, the chronology and results of which are summarized in Table 2 and Table 3. In August 2019, a routine electrocardiogram (ECG) was performed, showing atrial fibrillation (AF) at 120 bpm, initiating treatment with digoxin, diltiazen, and low molecular weight heparin (LMWH). Examined by the cardiology service, an echocardiogram was performed, which showed multiple valve disease, adjusting the treatment (Table 1).

Assessed in August 2020, in neurology outpatient clinics in relation to a double episode of nocturnal disorientation, a cranial CT scan was requested that found a “small cerebellar hemorrhage” requiring hospitalization for neurological surveillance. Treatment with edobaxan is preventedly suspended due to its anticoagulant properties. A brain study is completed with MRI that does not clearly show the presence of hemorrhage, ruling out malformations or other lesions that cause bleeding. There was good evolution during the hospital stay. A control cranial CT scan was performed that showed a punctiform image in the right cerebellar hemisphere corresponding to calcification, so the patient was discharged and the edobaxan regimen was restarted.

During the COVID-19 pandemic, a SARS CoV-2 antigen screening was performed (November 2020) with a negative result.

### 2.6. Follow-Up during 2021

In December 2020, the patient went to the new FM service of the center showing a defective speech related to her visual difficulties, including a negativistic discourse with complaints as well as a nihilistic view of her circumstances and her future. She also maintained her heart disease, brain damage, anxiety-depressive symptoms, side effects of drug treatment, and secondary functional dependence. The clinical examination showed a temperature of 35.7 °C, heart rate of 70 bpm (atrial fibrillation), blood pressure of 150/75 mmHg, and O_2_ saturation of 96%, resulting in normal physical and neurological examination. She reports complex visual hallucinations (people, animals, and objects) in the absence of cognitive impairment that appears to be Charles Bonnet syndrome.

During the months of January and June 2021, she attended four times for analytical control, assessment of the evolution and therapeutic adjustment (see Table 1, Table 2 and Table 3), in coordination with her cardiologist and her psychiatrist. Different analytical parameters have been requested including hemogram, proteinogram, kidney function tests, and glomerular filtration. Hepatopancreatic, ionogram, markers of heart failure such as NT pro-BNP, iron metabolism, and anemias screening have shown data suggestive of normality.

The SARS-COVID-19 immunization is carried out between the months of March and April 2021.

In consultation with her FM and carried out in October 2021, the patient attends in the company of her husband; she is very cooperative, smiling, and showing emotional stability, with absence of parasuicidal ideation and Charles Bonnet syndrome, which she associates with increased physical activity and psychotherapeutic as well as to correct pharmacological control, despite the fact that anxiety levels remain high, referring to fear of loss of family support (the results are shown in Table 1, Table 2 and Table 3).

## 3. Case Management from Family Medicine

Since it is a patient who lives in the community, the FM department of the health center has acted, coordinating the needs of monitoring of the different pathologies that she presents with the support of her family as a basic element of well-being. It is a classic FM strategy, implemented with the aim of achieving primary, secondary, and tertiary prevention.

## 4. Discussion

In the present case, the following areas of deliberation are raised: 1. Multifactorial etiology of the disease; 2. Diagnostic certainty; 3. Efficacy of psychopharmacological treatment; and 4. Role of the family in the patient’s care.

### 4.1. Multifactorial Etiology of the Disease

The main risk factors for depressive disorder in old adults have been frequently studied and include psychosocial circumstances of the aging process, personality factors, previous psychiatric pathology, intercurrent illnesses, and the interactions of associated treatments, although the level of influence of each factor is difficult to discriminate [18,19].

The present case could constitute a paradigm of the multicausality of depressive disorder in old adulthood, since, in a progressive and continuous way, several of the main factors associated with depressive symptoms that contribute to chronicity have been presented. In the psychosocial level, losses and grief, loneliness, environmental changes, and maladjustment stand out as potential etiological factors [20]. In this case, she is a person with a high cultural level, economic resources, comfortable habitat, and very stable social and family support. Regarding personality factors, some authors suggest that traits such as neuroticism increase the risk of presenting depressive symptoms in old adulthood [21]. It was not considered necessary to assess personality factors in a structured way, since an evolution of 10 years and the previous therapeutic approaches seem to be advisable, although it is true that the patient frequently refers to “a change in personality, from shyness to a certain disinhibition in the last years” associated with the general clinical picture that could be the result of antidepressant treatment. In the medical history, no references to previous psychiatric pathologies, consumption of toxic substances, or adjustment disorders were observed, with a stable work environment until her retirement.

Different studies associate metabolic diseases such as hypothyroidism and diabetes mellitus, or cardio and cerebrovascular disease, with an increased risk of suffering from depression, relating it to the multiple neuroimmunoendocrine changes in depressive patients. It has been observed that patients with depressive symptoms experience increased platelet activation that could predispose them to thromboembolic episodes. They also experience immune activation (NK cells and leukocytes) and hypercortisolemia, along with an increased adrenocorticotropic hormone (ACTH) and ACTH-releasing factor. In addition, they experience decreased insulin resistance, increased endogenous production of steroids, and the release of catecholamines, associated with an increase in arterial pressure and coronary vasoconstriction. Moreover, depressive symptoms constitute a poor prognostic factor in cardiovascular and metabolic diseases [22,23,24,25]. In this case, the protocol-based examinations showed no alteration justifying the role of physical factors in the depressive simptomatology. On the other hand, the polypharmacy used to control these diseases constitutes a known precipitating factor of depressive symptoms in old adults. Thus, drugs such as digoxin, diuretics, oral antidiabetics, and antihypertensives have been frequently associated with a greater risk of depression in these populations [26]. We cannot determine the level of influence of these drugs on the prognosis, but we can consider that their interactions with antidepressant drugs could make remission of the depressive symptomatology difficult.

In the clinical evolution of the patient, we consider the loss of vision to be key in the chronification of depressive symptoms due to the psychological repercussions as a factor of anxiety, insecurity, and fear; the functional repercussions for the instrumental and basic activities of daily life that limit self-care and potentiate iatrogenic risks; and the social repercussions related to leisure activities and increased consumption of resources, all of which favor frailty and limit self-perception of health status.

On the other hand, we consider the presence of a Charles Bonnet syndrome characterized by hallucinations to be of interest, which are commonly perceived as real by the patients and are related to visual deficits. Although the underlying mechanism is not well understood, it seems to be related to a brain’s continuous adjustment to significant vision loss. Old adults affected with Charlet Bonnet syndrome can avoid reporting to their doctor because of fear that the hallucinations could be related to a severe mental disorder. The clinical management consists of health education, explaining to the patient the nature of the disorder, the prevalent symptomatology, making them aware of the symptoms, and explaining that it is part of their visual deficit and not relevant to their depressive symptomatology. Eventually, pharmacological treatment with neuroleptics, benzodiazepines, antidepressants, and antiepileptics is required [16,17].

### 4.2. Diagnosis of Depression

As a complex diagnosis, major depression in old age involves assessing cognitive functions, behaviors, and the impact of any affective disorder on the functional capacity and quality of life of the patient. Following the DSM-5 criteria [11] facilitates the discrimination between a depressive disorder and a mixed adjustment disorder that could be better explained according to the current situation of the process. For the diagnosis of major depressive disorder, the criterion of temporality greater than two weeks, the presence of a depressed mood most of the day, and anhedonia, or a marked decrease in interest or a displeasure in almost all activities, are included; in addition, the presence of at least five additional symptoms are included, such as insomnia, hyporexia, loss of energy, inappropriate feelings of guilt and worthlessness, and self-destructive ideation, among others. In the case of mixed adjustment disorder, the diagnostic criteria include five groups (A–E), so that the anxiety-depressive symptoms occur in response to an identifiable stressor or factors that occur in the following three months. At the beginning of the stressor (A), the symptoms are clinically relevant with an intense and disproportionate discomfort in relation to the intensity of the stressor, generating a significant deterioration of social functioning or of other areas (B), other mental disorders are excluded (C), the symptoms do not represent a normal grief (D), and once the stressful event or its consequences have ended, the symptoms do not persist for more than another six months (E). In the case reported, it is not possible to fulfill criterion E because the most significant stress factors, those that generate the most discomfort and maladjustment, have become chronic so their resolution is not possible. Structured cognitive assessment has not been carried out because of the absence of progressive decline.

### 4.3. Efficacy of the Psychopharmacological Treatment

As has been reported, the pharmacological approach in this case is highly complex. Until the advent of SSRIs, the treatments of choice were TCA and tetracyclic (ATTC) antidepressants, but the potential induction of anticholinergic effects can cause cardiovascular alterations (orthostatic hypotension, arrhythmias, electrocardiographic alterations), changes in intestinal motility (constipation, paralytic ileus), urinary retention, and pupillary dilatation, among others, discouraging their use. Currently, SSRIs and SSNRIs are the dominant pharmacological approaches for depression in old adults, motivated by their ease of use, versatility, efficacy, and safety, in addition to their cost-effectiveness.

SSRIs (fluoxetine, fluvoxamine, paroxetine, sertraline, citalopram, escitalopram) work by blocking the reuptake of serotonin (5-HT) through inhibition of the adenosine triphosphatase (ATPase)-dependent sodium/potassium transporter (NA+/K+) in presynaptic neurons. With some differences between them, they have effects on other neurotransmission systems such as noradrenergic or dopaminergic. They are metabolized by liver enzymes, especially cytochrome P450 2D6, and have different pharmacokinetic characteristics. The main indication is major depression, although they are also useful in conditions such as obsessive-compulsive disorder or anxiety disorders. The most frequent side effects are gastrointestinal (nausea, burning, diarrhea), related to intestinal 5-HT receptors, which are minimized with a staggered dosage of medication. A variable percentage of patients treated with SSRIs manifest a sensation of activation of the central nervous system with agitation, nervousness, and insomnia that usually responds to moderate doses of benzodiazepines, such as alprazolam, lorazepam, or clonazepam. In general, they present a moderate risk of pharmacological interactions, and they are very safe drugs, as studies of lethal overdose show [27,28,29,30,31,32].

SSNRIs, such as duloxetine and venlafaxine, are a second group of drugs especially useful in the treatment of depression in old adulthood. SSNRIs may have a faster onset of action than other antidepressants by modulation of beta-adrenergic receptors. Duloxetine is a potent 5-HT and norepinephrine reuptake inhibitor with low affinity for muscarinic or histamine receptors, whereas venlafaxine shares 5-HT reuptake potency with moderate effects on norepinephrine reuptake and few effects on other neurotransmission systems. In addition, they present a low risk of pharmacokinetic interactions due to low potency in the inhibition of liver enzymes of cytochrome P450, a factor that facilitates their use. The FDA indications for this group of drugs are major depression, generalized anxiety disorder, and social anxiety disorder. Since they have a mechanism of action similar to that of tricyclic antidepressants, SSNRIs have shown their usefulness in some pain disorders, which makes them especially useful in older patients and in depression associated with neuropathic comorbidity. They share some of the gastrointestinal side effects with SSRIs, however, they differ from these in the moderate risk of increased blood pressure, somewhat less frequently in prolonged-release venlafaxine, which requires periodic monitoring in the first months of treatment and is solved by adjusting the dose. Other side effects described are syncope, ortostatic hypotension, and anticholinergic symptoms, such as dry mouth, urinary retention, and constipation, which in old patients must be monitored. Exceptional cases of fatal overdose have been described. Its level of efficacy compared to SSRIs seems somewhat higher, even though the data are discrepant [33,34,35,36,37,38].

In recent years, the approval of mirtazapine for the treatment of depression has led to its frequent use in old adults. It is an antagonist of alpha 2-adrenergic receptors that acts by increasing the release of norepinephrine that achieves a rapid increase in 5-HT levels, achieving modulation of the serotonergic system. Mirtazapine is metabolized through cytochrome P450 enzymes without being an inducer or inhibitor of these enzymes, so there are no interactions with other psychotropic agents, which facilitates the combination. Its main indication is major depression, used alone or in association with SSRIs/SSNRIs. Compared with paroxetine, it showed a faster response and fewer dropouts associated with adverse effects. Among the most frequent side effects are drowsiness (which advises its use at night) and increased appetite and weight. Furthermore, it seems to increase the levels of cholesterol and triglycerides secondarily, which, associated with its potential cardiovascular effect, makes it necessary to monitor blood pressure [39,40,41].

The therapeutic strategy is of great interest in this case. Since it is a highly complex case, the management of psychotropic drugs had to be careful, requiring consideration not only of the efficacy and probability of remission, but also of the minimization of secondary organic complications, to guarantee safety. In addition, the progressive appearance of comorbid, cardio, and cerebrovascular factors has required pharmacological adjustment. The potential interactions of the treatment must be considered, with the aim being its optimization. We consider the combined use of venlafaxine and mirtazapine to be successful due to its efficacy and safety, as evidenced by the adequate adherence of the patient to treatment and medical controls. In the case of duloxetine, its potential modification of blood pressure levels could question its use in this case [42]. The Goldberg Anxiety and Depression Scale (GADS) carried out in October 2021 suggests a remission of depressive symptoms and an improvement in the patient’s attitude. However, based on the results of the interview and the GADS (A7/D0), the use of benzodiazepines to control anxiety and insomnia symptoms does not seem clear.

### 4.4. Role of the Family in the Patient’s Care

This case presents many of the specific challenges in managing geriatric patients in the Galician health care model (northwest Spain). The guarantee of citizens’ health rights has been defined in the Spanish constitution since 1978. However, in 2002 there was a decentralization of competences in different areas, including health, according to the Law of Cohesion and Quality of the National Health System, which established a framework in the 17 autonomous communities of the Spanish State, but with peculiarities according to each territory. Regardless of this framework, the citizens, using their freedom, choose in each health situation whether to be treated in the public health system (in Galicia, the Servicio Galego de Saúde, or SERGAS) or in the free market system (private clinical services companies and/or consultations by private professionals), or both. The reality is that this mixed system can condition the efficiency of geriatric and psychiatric interventions in complex cases, hindering actions from primary health care because decision-making is dispersed. In this context, relevant information for therapeutic optimization is frequently lost.

The socioeconomic context of the patient allows clinical follow-up with good health resources, within a dual system (private and public) that contributes to effective health care, although its efficiency is limited by the heterogeneity of clinical opinions. As it has been mentioned above, the health care model in Galicia is based on a public, universal system in coexistence with private companies and entities of the social sector that provide health and care services, in addition to freelance professionals in health areas such as ophthalmology, psychiatry, internal medicine, or psychology, among many others. Between 2020 and 2021, the COVID-19 pandemic has required the adoption of restrictive measures in terms of prevention and mobility attitudes that seem to increase the incidence of psychiatric pathology in old populations [43,44], although in this case it does not seem to have conditioned the evolution of the patient.

We believe that a more intensive non-pharmacological approach would contribute to improving the prognosis; specifically, it would reduce anxiety-type symptoms and achieve a more objective self-perception of health. It would be an area for improvement using some of the usual techniques in similar cases, from cognitive behavioral to supportive or family therapy. In recent years, third generation behavioral therapies seem to contribute to intervention in psychogeriatrics. These include Acceptance and Commitment Therapy, Dialectical Behavioral Therapy, Mindfulness-Based Therapy, Behavioral Activation Therapy, Integral Behavioral Couple Therapy, or Functional Analytical Psychotherapy, which share an integrative vision of the psychological problems of old patients, considering their functional structure relevant, that is, the psychological functions of maladaptive behaviors in the context in which they occur [45]. These types of approaches may probably contribute to improve the quality of life and the health perception of the patient.

The evolution of the depressive disorder is linked to the role that her husband plays in psychological care and functional support for her activities of daily living. The long duration of the disease and the appearance of associated pathologies increase the intensity of care. The parallel aging of the husband and the incidence of medical and psychological problems could contribute to a potential claudication or the development of caregiver burnout [46,47].

## 5. Conclusions

The present work discusses the complexity of the diagnosis and treatment of depression in the geriatric patient. It is illustrated with the case of a patient (a 75-year-old woman) with depressive symptomatology with more than 10 years of evolution, also affected by different concomitant organic pathologies including visual deficits and Charles Bonnet syndrome. The interventions of different medical specialties are shown, and some psychopharmacological treatment options are discussed. The interactions of the different pharmacological treatments and the mixed care approaches are considered, with the aims of improving the case management and maximizing the quality of life of the patient in this type of complex clinical condition. The complexity of the healthcare system in Galicia (northwest Spain) and how difficult it is to handle complex geriatric cases in this context are discussed. In this regard, the most relevant limitation of this case in the lack of a specific approach, substituted for this patient by a mixed care model. Other limitations include the lack of a personality assessment using psychometrically valid tests, the lack of an explicit frailty assessment beyond the clinical observation of an increased bio-psycho-social vulnerability, and the lack of an objective assessment of the caregiver burden.

## Figures and Tables

**Table 1:** Evolutive drug adjustments.

Drugs/Year	2014	2018	2019	2020	2021-1	2021-2
Levotiroxina	100	100	100	100	100	100
Metformina	-	-	850	850	1275	1275
Espironolactona	25	25	25	-	-	-
Digoxina	-	-	125	125	125	125
Diltiazem R	-	-	120	120	120	120
Azetazolamida	-	-	250	250	250	250
Boi-K	-	-	1 c	1 c	1 c	1 c
Edobaxan	-	-	60	60	60	60
Armolipid/Lipok	’1 c	1 c	1 c	1 c	1 c	1 c
Ezetimiba	-	10	10	10	10	10
Atorvastatina	-	10	-	-	-	-
Duloxetina	60	60	-	-	-	-
Citalopram	-	-	10	-	-	-
Venlafaxin R	-	-	-	150	150	150
Venlafaxin	-	-	-	75	75	75
Mirtazapine	-	-	-	-	15	15
Lorazepam	1	1	1	1	1	1
Clonazepam	0.6	0.6	0.6	-	-	0.6

Note: 2021-1 (February 2021). 2021-2 (October 2021). Boi-K: Potassium hydrogen carbonate 1001 mg and ascorbic acid 250 mg, with dose in mgs. c: capsule.

**Table 2:** Control serum parameters.

Parameter/Year	2014	2018	2019	2020	2021-1	2021-2
TSH	4.22	niop	4.50	4.96	2.30	2.95
T3	8.2	niop	8.7	niop	7.5	6.5
T4	0.9	niop	0.9	niop	0.8	0.8
Vit D	niop	niop	niop	34.6	42.77	39.1
Glu	112	115	125	136	156	151
Hgb A1c	6.2	6.1	niop	niop	6,6	6.1
Cholesterol	288	224	240	155	164	171
HDL	58	47	41	43	49	52
LDL	214	162	150	90	82	100
Triglicéridos	80	77	81	110	165	93
Digoxinemia	niop	niop	niop	niop	niop	0.8

Note: 2021-1 (February 2021). 2021-2 (October 2021). Parameters in mg/dl. Hgb A1c in %. Digoxinemia in nanograms/mL. niop: not included or provided.

**Table 3:** Control cardiac and psychological paremeters.

Parameter/Year	2014	2018	2019	2020	2021-1	2021-2
SBP	120	135	116	125	150	135
DBP	65	66	67	70	75	70
Heart rate	80	80	120	80	70	72
SO%	niop	99	niop	niop	96	96
ECG	niop	niop	AF	AF	AF	AF
Test						
GADS	-	-	-	-	8/5	7/0

Note: 2021-1 (February 2021). 2021-2 (October 2021). AF (Atrial Fiblilation). GADS: Goldberg Anxiety and Depression Scale. niop: not included or provided.

## Data Availability

The data presented in this study can be requested to the corresponding author. The data are not publicly available due to confidentiality and anonymity.
